# Heat-Killed *Lactiplantibacillus plantarum* LRCC5314 Mitigates the Effects of Stress-Related Type 2 Diabetes in Mice via Gut Microbiome Modulation

**DOI:** 10.4014/jmb.2111.11008

**Published:** 2021-12-17

**Authors:** YoHan Nam, Seokmin Yoon, Jihye Baek, Jong-Hwa Kim, Miri Park, KwangWoo Hwang, Wonyong Kim

**Affiliations:** 1 Department of Microbiology, College of Medicine, Chung-Ang University, Seoul 06974, Republic of Korea; 2 College of Pharmacy, ^2^Chung‐Ang University, Seoul 06974, Republic of Korea; 3 Lotte R&D Center, Seoul 07594, Republic of Korea

**Keywords:** Stress-T2D, *Lactiplantibacillus plantarum*, postbiotics, heat-killed, microbiome

## Abstract

The incidence of stress-related type 2 diabetes (stress-T2D), which is aggravated by physiological stress, is increasing annually. The effects of *Lactobacillus*, a key component of probiotics, have been widely studied in diabetes; however, studies on the effects of postbiotics are still limited. Here, we aimed to examine the mechanism through which heat-killed *Lactiplantibacillus plantarum* LRCC5314 (HK-LRCC5314) alleviates stress-T2D in a cold-induced stress-T2D C57BL/6 mouse model. HK-LRCC5314 markedly decreased body weight gain, adipose tissue (neck, subcutaneous, and epididymal) weight, and fasting glucose levels. In the adipose tissue, mRNA expression levels of stress-T2D associated factors (NPY, Y2R, GLUT4, adiponectin, and leptin) and pro-inflammatory factors (TNF-&alpha;, IL-6, and CCL-2) were also altered. Furthermore, HK-LRCC5314 increased the abundance of *Barnesiella*, *Alistipes*, and butyrate-producing bacteria, including *Akkermansia*, in feces and decreased the abundance of *Ruminococcus*, *Dorea*, and *Clostridium*. Thus, these findings suggest that HK-LRCC5314 exerts protective effects against stress-T2D via gut microbiome modulation, suggesting its potential as a supplement for managing stress-T2D.

## Introduction

Probiotics are non-pathogenic, live microorganisms that are widely used worldwide [1, 2]. Currently, there is a growing awareness that probiotics can be used as functional foods and dietary supplements because of their potential effects on human health, such as immune modulation, cholesterol reduction, obesity amelioration, and reduction in the risk of many other diseases [3, 4]. However, there are limited assessments of probiotics due to the restricted regulation of probiotics and lack of adequate treatment for specific clinical situations [3, 5].

Postbiotics are more profitable than probiotics because of their safety, long shelf life, and ease of transportation and storage [5, 6]. Postbiotics, derived from live probiotics, are defined as a combination of their metabolites and bioactive compounds. These postbiotics include tyndallized and heat-killed forms and can be acquired using various methods, including heat, ultraviolet rays, chemicals, and sonication [7]. Many studies related to postbiotics have been performed to explore their beneficial properties in cells, animals, and clinical trials [8-10]. The heat-killed form shows beneficial effects such as stimulation of the immune system in various diseases and modulation of the gut environment, which regulates anti-inflammatory, anti-obesogenic, and anti-hypertensive activities [11, 12].

The incidence of stress-related type 2 diabetes (stress-T2D), which affects the quality of life, has increased annually, and it is expected that approximately a fifth of the global population will experience stress-T2D by 2025 [13, 14]. Cold stress is a form of physiological stress elicited by the stress-response center, namely, the hypothalamic–pituitary–adrenal (HPA) axis [15]. In addition, cold stress involved in insulin signaling is used to induce metabolic syndromes such as diabetes [16].

Corticosterone is released in response to stress and affects energy homeostasis, lipid metabolism, glucose metabolism, insulin sensitivity, and immunological functions [17-19]. Moreover, these actions induce a biological response, which can lead to type 2 diabetes (T2D) [20].

However, research on the relationship between chronic physiological stress and biological process in T2D has only been conducted using animal models, and human studies are very limited [21]. In addition, most studies on stress-T2D are based on observational data, and the conclusions remain unclear [20]. Most stress-related studies have focused on the effects of probiotics rather than postbiotics [22].

Trillions of bacteria in the gut regulate metabolism, digestion, vitamin synthesis, immunomodulation, and immunity against pathogens [23]. Furthermore, the gut microbiome is associated with stress and stress-related disorders and regulates the secretion of inflammatory cytokines by immune cells [24, 25].

This complex, multifaceted microbial system is affected by behavior, physiology, stress responsiveness, and anxiety [26]. Even in cases of short-term exposure to stress, the microbiota community can be influenced by changes in the relative proportions of the major microorganisms [27]. Moreover, several studies related to the function of the microbiome described the beneficial effects of prebiotics and probiotics on mental health using animal models [28].

The genus *Lactobacillus* has been reclassified into 25 genera based on whole-genome sequences [29]. *Lactobacillus plantarum* was therefore reclassified into *Lactiplantibacillus plantarum*. In this study, we sought to characterize the potential effects of the postbiotic heat-killed *L. plantarum* LRCC5314 (HK-LRCC5314) on stress-T2D by analyzing the gut microbiota composition and stress-T2D-associated factor modulation in an animal model.

## Materials and Methods

### Animal Experiment

Nine-week-old C57BL/6J male mice (*n* = 24) were purchased from the Central Lab Animal Incorporation (Korea) and used in this experiment according to the guidelines of the Chung-Ang University Institutional Animal Care and Use Committee of the Laboratory Animal Research Center (IACUC No. 2018-00022). All experiments were approved by the Korean Food and Drug Administration [30].

To induce cold stress in mice, each group of mice was directly held on ice for 1 h. To induce T2D, all experimental groups were fed a high-fat diet (HFD; 60% of calories from fat), which was purchased from Central Lab Animal Incorporation. These conditions were maintained until the end of the experimental period (12 weeks). At 12 weeks, the mice were sacrificed via CO_2_ inhalation and adipose tissues (neck, subcutaneous, and epididymal) and sera were collected for further analysis.

The mice were randomly divided into three groups: (1) control group, stress-T2D treated with phosphate-buffered saline (PBS; *n* = 8); (2) placebo group, stress-T2D treated with *L. gasseri* BNR 17 (approximately 1 × 108 CFU/day; *n* = 8); and (3) HK-LRCC5314 group, stress-T2D treated with HK-LRCC5314 (approximately 1 × 108 CFU/day; *n* = 8). All experimental groups were administered with 200 μl of the indicated treatment for 12 weeks. The BNR 17 and HK-LRCC5314 powder samples were reconstituted in PBS. *L. gasseri* BNR17 is a probiotic product commercially available in Korea, which suppresses weight gain in obesity and T2D [31, 32]; thus, it was used for comparison with HK-LRCC5314.

### Microbiota Genomic DNA Extraction and Metagenomic Sequencing

Total genomic DNA of the microbiota in the mouse fecal samples at 12 weeks after administration was extracted using the FastDNA Spin Kit for Soil (MP Biomedical, USA) according to the manufacturer’s instructions. The V3–V4 region of the 16S rRNA gene was amplified using the bacterial primer set 347F/803R. Sequencing libraries were developed using the Illumina MiSeq System (Macrogen, Inc., South Korea). Raw reads were processed using the Quantitative Insights Into Microbial Ecology software (QIIME2, version 2021.2) with quality trimming. Demultiplexed sequences were imported as artifacts and denoised using DADA2 [33]. In this step, chimera sequence filtering was performed to ensure the maintenance of high-quality reads. Amplicon sequence variants were processed for taxonomic and diversity analyses. Taxonomic assignment of individual datasets was performed using the Green Genes database (gg-13_8 99%) [34].

### Analysis of Serum Corticosterone Concentration

Mouse serum samples were collected to determine the corticosterone concentration using an appropriate ELISA kit (R&D Systems, Germany), according to the manufacturer’s guidelines. Absorbance was measured at 405 nm using a NanoQuant spectrophotometer (Infinite 200; Tecan Inc., Switzerland).

### Fasting Glucose Level and Glucose (GTT) and Insulin (ITT) Tolerance Tests

Mice were fasted for 16 h with access to drinking water, and the blood glucose levels were measured once per week. In addition, GTT and ITT were performed to assess glucose metabolism and insulin sensitivity, respectively, caused by stress and T2D. For the GTT, the mice were fasted for 16 h with access to drinking water. For the ITT, mice were fasted for 6 h. For the GTT, 20% D-glucose (Sigma-Aldrich, USA) solution per body weight was administered intraperitoneally. For the ITT, 0.1 U/ml insulin (SAFC Biosciences Inc., USA) per body weight was administered intraperitoneally. Blood was collected from the tail vein to measure glucose levels. Abnormal behavior was carefully observed at 0, 15, 30, 60, and 120 min after administration of glucose/insulin.

### Analyses of Body and Adipose Tissue Weight

The body weights of the mice were measured once per week. Mouse adipose tissue (neck, subcutaneous, and epididymal) were weighed immediately after euthanasia. The adipose tissues were harvested, rinsed with PBS, and stored for further analysis.

### mRNA Expression Analysis Using Real-Time Polymerase Chain Reaction (PCR)

Epididymal adipose tissues were used to assess the expression of mRNAs related to stress [neuropeptide Y (NPY) and Y2-receptor (Y2R)], obesity [adiponectin, leptin, and glucose transporter type 4 (GLUT4)], and inflammation [tumor necrosis factor (TNF-α), interleukin (IL)-6, and chemokine ligand 2 (CCL2)]. Total RNA was extracted using the RNeasy Mini Kit (Qiagen, Germany) and eluted in nuclease-free water. The concentration of the extracted RNA was determined using a NanoQuant spectrophotometer (Infinite 200, Tecan Inc.,). cDNA was synthesised from 0.5 μg of extracted RNA using a PrimeScript 1st Strand cDNA Synthesis Kit (Takara Bio, Japan). The difference in mRNA expression was quantified using the 7500 Fast Real-time PCR system (Applied Biosystems, USA) and the SYBR Green PCR Kit (Qiagen), and the fold change was calculated using the 2^−ΔΔCt^ method. The control gene 18S rRNA was used as a reference.

### Statistical Analyses

Results are presented as the mean ± SEM. The data were normally distributed, and significant differences between groups were determined using an unpaired *t*-test and two-way analysis of variance (ANOVA). Statistical significance was set at *p* < 0.05. All analyses were performed using GraphPad Prism version 6.0. and 8.0. To compare the relative differences in the microbiota abundance among groups, statistical results were analyzed using the R software (version 4.0.2) and GraphPad Prism software with Wilcoxon and Kruskal–Wallis tests. Alpha diversity was determined based on the Shannon and Simpson indices, whereas beta diversity was determined by assessing nonmetric multidimensional scaling (NMDS) of Bray–Curtis distance using the vegan package [35]. Taxonomic composition was determined using the DESeq2 [36] and phyloseq packages [37]. Furthermore, comparative statistical analyses among the groups were performed using two-way ANOVA and Tukey–Kramer post-hoc tests using the Statistical Analysis of Metagenomic Profiles (STAMP) software [38]. The raw Illumina read data are deposited in the NCBI Sequence Read Archive under the accession numbers SRR14089764–SRR14089785.

## Results

### Effects of HK-LRCC5314 Intake on Microbiota Composition in the Cold Stress-Induced T2D Mouse Model

**Fig. 1:**
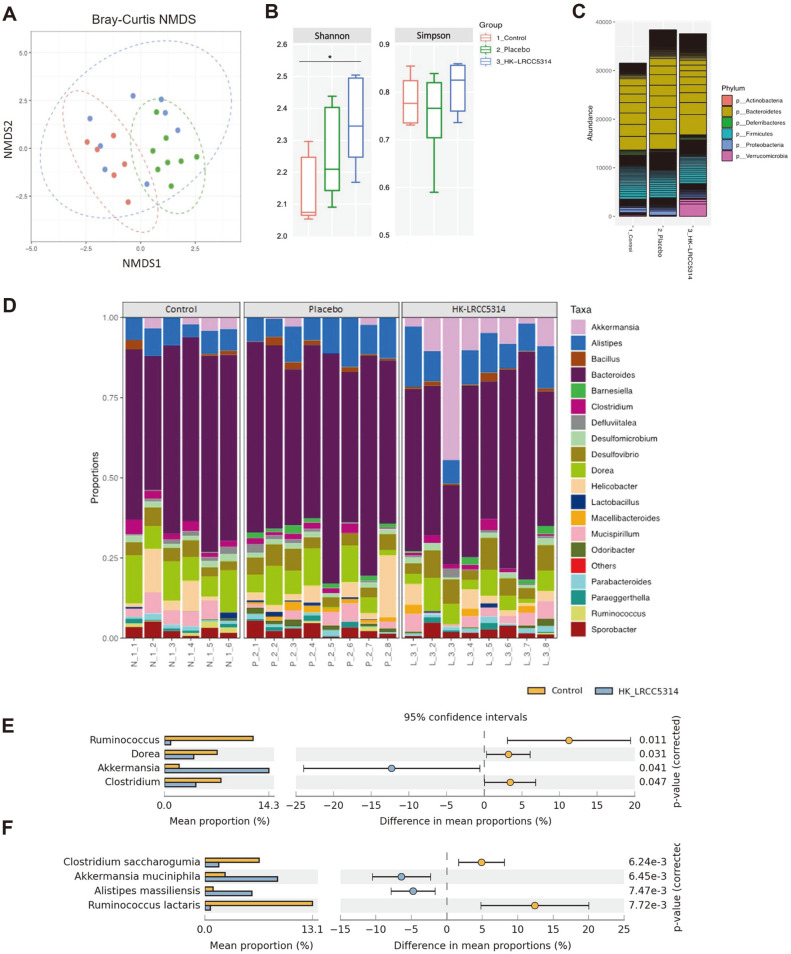
Effect of postbiotic HK-LRCC5314 on the composition of the gut microbiota. (**A**) Nonmetric multidimensional scaling ordination method based on the Bray–Curtis dissimilarity matrix. (**B**) Alpha diversity box plots according to the Shannon and Simpson indices. Bar chart showing the gut microbiota composition at the (**C**) phylum and (**D**) genus levels. Distinct abundances at the (**E**) genus levels observed in the control and HK-LRCC5314 groups. *p < 0.05 vs control.

A total of 2,080,250 sequence reads was obtained from 22 mice (average: 93,117 ± 9,814). The NMDS ordinations based on Bray–Curtis distance showed that the gut microbiota was significantly diverse among the groups (Fig. 1A; PERMANOVA: *F* = 2.94, *p* < 0.001). For alpha diversity, a slightly increased alpha diversity based on Simpson’s index was observed in the HK-LRCC5314 group, although the difference was not statistically significant (*p* = 0.05). However, a significant increase in the diversity based on the Shannon index was observed in the HK-LRCC5314 group (*p* = 0.026; Fig. 1B). Moreover, the relative abundance at the phylum and genus levels differed among the groups (Figs. 1C and 1D). Although several bacterial groups were present in all groups, their relative abundances were significantly different between the control and HK-LRCC5314 groups. The phyla Verrucomicrobia and Bacteroidetes showed relatively high abundances in HK-LRCC5314 group. At the genus level, significant enrichments of *Akkermansia* (phylum Verrucomicrobia) and *Barnesiella* and *Alistipes* (all belonging to phylum Bacteroidetes) were observed in the HK-LRCC5314 group. However, *Ruminococcus*, *Dorea*, and *Clostridium* (all belonging to phylum Firmicutes) were more abundant in the control (Fig. 1E).

### Effects of HK-LRCC5314 Administration on Corticosterone Concentration in the Cold Stress-Induced T2D Mouse Model

**Fig. 2:**
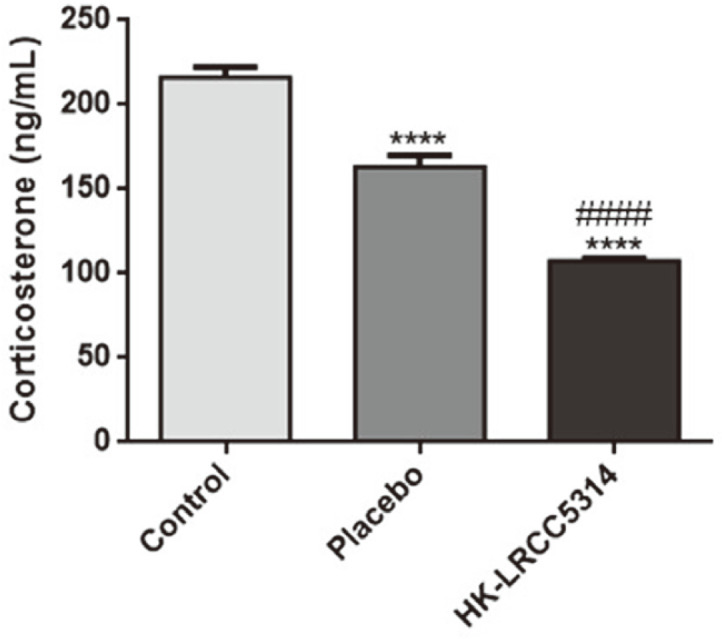
Effects of postbiotic HK-LRCC5314 on serum corticosterone levels under stress-related T2D conditions. The corticosterone levels were assessed using ELISA. ****p < 0.0001 vs. control and ^####^*p* < 0.0001 vs. placebo.

The highest serum corticosterone concentration was observed in the control group (Fig. 2). Corticosterone levels in the HK-LRCC5314 group were significantly lower than those in the control group (*p* < 0.0001). Furthermore, treatment with HK-LRCC5314 showed lower levels of corticosterone than in the placebo group (*p* < 0.0001).

### Effects of HK-LRCC5314 Intake on Fasting Glucose Level and GTT/ITT Results in the Cold Stress-Induced T2D Mouse Model

**Fig. 3:**
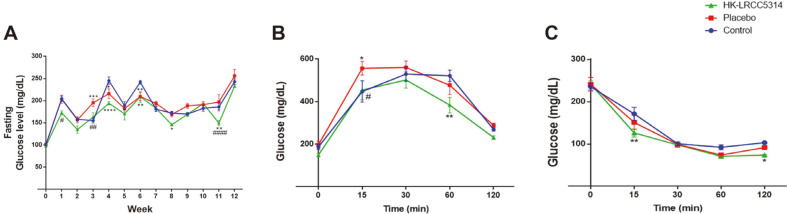
Effects of postbiotic HK-LRCC5314 treatment on fasting glucose level by performing glucose (GTT) and insulin (ITT) tolerance tests. (**A**) The fasting glucose level was measured using the tail vein blood sample. (**B**) GTT and (**C**) ITT were performed following intra-peritoneal injection. *p < 0.05, **p < 0.001, ***p < 0.0005, ****p < 0.0001 vs. control. ^#^*p* < 0.05, ^##^*p* < 0.001, ^####^*p* < 0.0005, ^####^*p* < 0.0001 vs. placebo.

Fasting glucose levels were determined each week, and various changes in glucose levels were observed during the experimental period. In general, treatment with HK-LRCC5314 showed similar or lower fasting glucose levels every week compared to those in the control and placebo groups. In the final week, the lowest glucose level was observed in the HK-LRCC5314 group when compared to the control (*p* = 0.6186) and placebo groups (*p* = 0.0788)(Fig. 3A).

At the starting point (0 min) after administration of glucose/insulin, glucose levels showed no difference between the groups in GTT and ITT. During the GTT, the glucose levels continued to increase from 0 to 30 min and decreased after 30 min in the HK-LRCC5314 group; however, in the placebo group, glucose levels decreased after 60 min (Fig. 3B). Treatment with HK-LRCC5314 yielded lower glucose levels than in the control (*p* = 0.0052) and placebo groups (*p* = 0.0823) at 60 min. Furthermore, the HK-LRCC5314 group showed the lowest glucose levels at 120 min. However, the glucose levels were not significantly different between the control (*p* = 0.6540) and placebo (*p* = 0.3864) groups. During the ITT, glucose levels continued to decrease until 60 min in all groups (Fig. 3C). At 15 min, the HK-LRCC5314 group showed lower glucose levels than those in the control (*p* = 0.0011) and placebo groups (*p* = 0.1137). At 120 min, treatment with HK-LRCC5314 showed lower glucose levels than those in the control (*p* = 0.0484) and placebo groups (*p* = 0.3269).

### Effects of HK-LRCC5314 Administration on mRNA Expression in the Cold Stress-Induced T2D Mouse Model

**Fig. 4:**
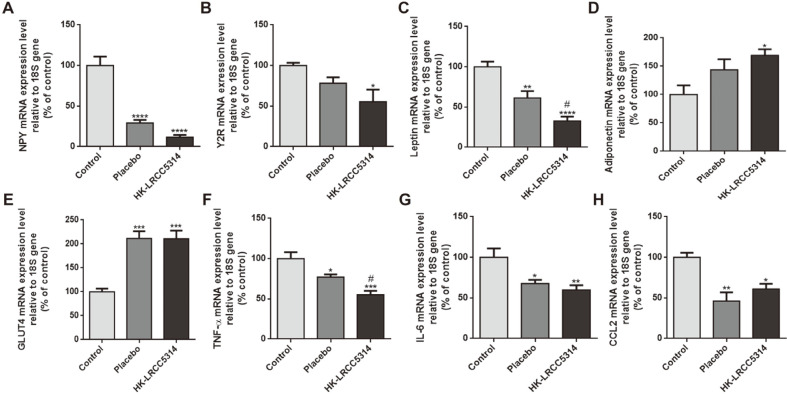
Effects of postbiotic HK-LRCC5314 treatment on the mRNA expression of stress-related, obesityrelated, and pro-inflammatory factors in epididymal adipose tissues. Stress-related factors: (**A**) NPY and (**B**) Y2R; obesity-related factors: (**C**) leptin, (**D**) adiponectin, and (**E**) GLUT4; and pro-inflammatory factors: (**F**) TNF-α, (**G**) IL-6, and (**H**) CCL2. *p < 0.05, **p < 0.001, ***p < 0.0005, ****p < 0.0001 vs. control. ^#^*p* < 0.05, ^##^*p* < 0.001, ^####^*p* < 0.0005, ^####^*p* < 0.0001 vs. placebo.

NPY mRNA levels were significantly decreased in the HK-LRCC5314 group compared to those in the control (*p* < 0.0001) and placebo groups (*p* < 0.0001). Moreover, NPY mRNA levels were lower in the HK-LRCC5314 group than in the placebo group (*p* = 0.1638), but the difference was not statistically significant. Moreover, the Y2R mRNA levels of the HK-LRCC5314 group were lower than those of the control (*p* = 0.0187) and placebo groups (*p* = 0.2181). There was a significant difference in the control but no significant difference in placebo (Figs. 4A-4B) as compared to the HK-LRCC5314 group.

Furthermore, leptin mRNA levels were lower in the HK-LRCC5314 group than in the control (*p* < 0.0001) and placebo groups (*p* = 0.0246). In the HK-LRCC5314 group, higher adiponectin mRNA levels were observed than those in the control (*p* = 0.0207) and placebo (*p* = 0.4407) groups (Figs. 4C-4D). In addition, insulin-regulated GLUT4 mRNA levels were higher in the HK-LRCC5314 group than in the control group (*p* = 0.0005) (Fig. 4E). Pro-inflammatory factors (TNF-α, IL-6, and CCL2) showed lower mRNA levels in the HK-LRCC5314 group. Specifically, TNF-α mRNA levels were considerably decreased compared to those in the control (*p* = 0.0003) and placebo groups (*p* = 0.0349). IL-6 mRNA levels were lower in the HK-LRCC5314 group than in the control group (*p* = 0.0081). Similarly, CCL2 mRNA levels were lower in the HK-LRCC5314 group than in the control group (*p* = 0.0218) (Figs. 4F-4H).

### Effects of HK-LRCC5314 Intake on Body and Adipose Tissue Weight in the Cold Stress-Induced T2D Mouse Model

Before cold stress was induced, the initial body weights of the control, placebo, and HK-LCC5314 groups were similar (Table 1). Body weight increased weekly in all groups, but the HK-LRCC5314 group exhibited the lowest rate. Furthermore, the final body weights of all groups were different (control: 38.38 ± 0.62 g; placebo: 41.13 ± 0.72 g; HK-LRCC5314: 32.88 ± 0.46 g). Specifically, the HK-LRCC5314 group had the lowest body weight compared to that in the control (*p* < 0.0001) and placebo groups (*p* < 0.0001).

**Fig. 5:**
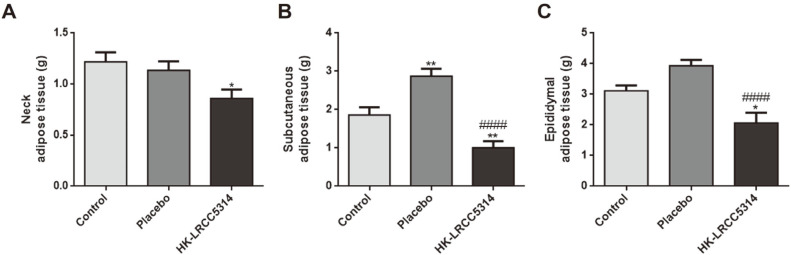
Effects of postbiotic HK-LRCC5314 treatment on the adipose tissue weight. (**A**) Neck, (**B**) subcutaneous, and (**C**) epididymal adipose tissues were weighed after the mice were euthanised. *p < 0.05, **p < 0.001, ***p < 0.0005, ****p < 0.0001 vs. control. ^#^*p* < 0.05, ^##^*p* < 0.001, ^###^*p* < 0.0005, ^####^*p* < 0.0001 vs. placebo.

The weights of neck adipose tissue in the HK-LRCC5314, control, and placebo groups were 0.85 ± 0.18 g, 1.21 ± 0.18 g, and 1.13 ± 0.18 g, respectively. These results showed that treatment with HK-LRCC5314 decreased the weight of neck adipose tissue compared to that of the control (*p* = 0.0198) and placebo groups (*p* = 0.0773)(Fig. 5A). Moreover, the weights of the subcutaneous adipose tissue in the HK-LRCC5314, control, and placebo groups were 1.01 ± 0.25 g, 1.86 ± 0.27 g, and 2.88 ± 0.26 g, respectively, showing that treatment with HK-LRCC5314 decreased the weight compared to that of the control (*p* = 0.0070) and placebo (*p* < 0.0001) groups (Fig. 5B). Additionally, the weights of epididymal adipose tissue in the HK-LRCC5314, control, and placebo groups were 2.06 ± 0.36 g, 3.11 ± 0.26 g, and 3.93 ± 0.26 g, respectively, indicating that treatment with HK-LRCC5314 decreased the weight compared to those of the control (*p* = 0.0134) and placebo (*p* < 0.0001) groups (Fig. 5C).

## Discussion

In this study we investigated the mechanism underlying the mitigation of stress-T2D by the postbiotic HK-LRCC5314 through its interacting with the gut microbiota and modulating lipid metabolism and immunomodulation in a C57BL/6J mouse model. In this study, the gut microbiota composition and alpha (Shannon and Simpson indices) and beta diversities (NMDS) were relatively different between the HK-LRCC5314 and control groups. A previous study reported a difference in gut bacterial diversity between patients with diabetes and healthy controls [39]. In addition, increased gut microbial diversity has been associated with improvements in the immune system [40]. Moreover, the phyla Verrucomicrobia and Bacteroidetes were more abundant in the gut microbiota of the HK-LRCC5314 group than in the control and placebo groups, as seen in this study. This finding is corroborated by the results of another study demonstrating that treatment with heat-killed *L. fermentum* and *L. delbrueckii* in mice induced microbial community changes, in which the proportion of Verrucomicrobia was increased in the gut microbiota [41] In addition, the proportion of Bacteroidetes was decreased in gut microbiota in T2D [42]. Therefore, HK-LRCC5314 may have a positive effect on the abundance of Verrucomicrobia and Bacteroidetes. Furthermore, the abundance of *Ruminococcus*, *Dorea*, and *Clostridium* was significantly decreased, whereas *Akkermansia*, *Barnesiella*, and *Alistipes* were significantly increased in the HK-LRCC5314 group when compared to the control group. Previously high levels of *Ruminococcus* and *Clostridium* were associated with T2D [43, 44]. In addition, several inflammatory cytokines were increased by *Ruminococcus* in T2D [45]. By contrast, *Akkermansia* led to alleviation of glucose intolerance, insulin resistance, adipose tissue inflammation, and enhancement of glucose tolerance in obesity and T2D [46] while also being associated with the production of butyric acid and reduction in gut permeability [47]. Moreover, *Barnesiella* and *Alistipes* abundances are associated with alleviation of inflammation and positive effects on obesity [48, 49]. They also have beneficial effects, such as mitigating insulin resistance, low inflammation in obesity and anti-diabetic effects [50, 51]. These results suggest that HK-LRCC5314 administration may recover gut microbiota diversity, increase the number of beneficial bacteria, and also decrease the number of T2D-related taxa.

Furthermore, HK-LRCC5314 treatment showed beneficial effects in suppressing body weight gain, lowering fasting blood glucose levels, and improving GTT and ITT results in T2D. Several studies have demonstrated that treatment with heat-killed *Lactobacillus* strains (*e.g.*, *L. plantarum* LN4 and *L. plantarum* L-137) can improve insulin sensitivity and downregulate glucose metabolism [52, 53]. On the other hand, our research demonstrated that treatment of BNR17 resulted in the increased body weight in stress-T2D. However, other studies demonstrated that BNR17 showed the beneficial effects of weight loss in obesity and T2D [54, 55]. In general, stresses that are regulated by HPA and corticosterone are related with body weight, but chronic stress may show weight gain and abdominal obesity [56]. Therefore, since our research was conducted in chronic cold-stress exposure, BNR17 may show increased body weight. The abundance of beneficial bacteria in the intestine is increased by probiotics, which changes the composition of the intestinal microbiota and has a potential beneficial effect against T2D by affecting energy and glucose metabolism and insulin sensitivity [57, 58]. These results suggest that HK-LRCC5314 treatment may have beneficial effects on the glucose metabolism of mice with stress-T2D.

In this study, HK-LRCC5314 treatment suppressed corticosterone secretion in mice. Corticosterone, a major hormone induced as a stress response and stress mediator, plays a key role in insulin resistance signaling, intestinal inflammatory response, and T2D via stressors [59, 60]. In addition, combination of chronic stress exposure and HFD showed increased corticosterone secretion accompanied by elevated HPA activity. These conditions were associated with alternation of intestinal bacterial composition and intestinal inflammatory markers [60]. Previous studies have demonstrated that heat-killed *L. casei* DKGF7 and *L. paracasei* treatment reduced serum corticosterone levels along with low inflammatory cytokines in intestine in murine models [61, 62]. Noteworthy in our study was that change in intestinal microbiota composition, secretion of low levels of corticosterone and alleviated inflammation were demonstrated by treatment with HK-LRCC5314 in stress-T2D.

Oral administration of HK-LRCC5314 also showed beneficial effects on the mRNA expression of factors related to obesity, glucose utility, stress, and inflammation. Previously, some *L. plantarum* strains showed effects of anti-obesity including glucose tolerance and inflammatory response induced by HFD rather than stress [63]. The mRNA levels of NPY and Y2R were significantly decreased by treatment with HK-LRCC5314. NPY is an endogenous neuropeptide associated with stress, cardiovascular physiology, anxiety, depression, and diabetes [64]. In addition, it is related to the HPA axis and is believed to be essential in stress regulation; it is also related to the expression of Y2R [65]. NPY-Y2R, which is stimulated by stress hormones in exposure to cold stress, induces adipocyte proliferation [66]. Mice treated with *L. casei* LC122 and *Bifidobacterium longum* BL986 showed decreased NPY mRNA expression [67]. Moreover, mRNA expressions of obesity-related factors (GLUT4 and adiponectin) were increased after HK-LRCC5314 treatment, whereas leptin levels were decreased. Stress exposure is related with glucose-insulin metabolism and leads to T2D [68]. GLUT4 mediates glucose uptake by stimulating insulin in adipose tissue [69]. Treatment with *L. rhamnosus* GG resulted in an increase in GLUT4 mRNA expression in the adipose tissues of HFD-fed mice [70]. Moreover, adiponectin is known to increase insulin sensitivity and support the expansion of adipose tissue [71]. However, decreased adiponectin levels are associated with diabetes. Likewise, leptin is associated with food intake, body weight, and energy expenditure. Leptin resistance is induced by HFD and is related to obesity and T2D [72]. *L. plantarum* HAC01 treatment resulted in increased adiponectin and decreased leptin mRNA expression in adipose tissues of an obese murine model [73], similar to the results of the present study. In addition, secretion of pro-inflammatory cytokines (TNF-α, IL-6, and CCL2) from macrophages and adipocytes is promoted in obesity, which is considered a form of chronic inflammatory disease [74]. Furthermore, cytokine secretion affects insulin signaling and lipid metabolism in overweight or obese individuals [75]. In this study, the levels of the pro-inflammatory factors were attenuated by HK-LRCC5314 treatment. A mixture of *L. plantarum* DSR M2 and *L. plantarum* DSR 920 was previously shown to alleviate the expression of pro-inflammatory cytokines in HFD-fed mice [76].

In conclusion, this study demonstrated the beneficial effects of HK-LRCC5314 by investigating cold stress-induced T2D in mice fed a HFD. HK-LRCC5314 treatment was found to induce changes in the gut microbiota during stress-T2D, demonstrating the possibility of a therapeutic effect on T2D via regulation of stress-T2D associated factors, including glucose metabolism. These results indicate that HK-LRCC5314 may be used as a potential functional supplement to alleviate T2D.

## Figures and Tables

**Table 1:** The initial and final body weight of each treatment group.

	Control	Placebo	HK-LRCC5314
Initial week	19.25 ± 0.40	19.50 ± 0.31	19.13 ± 0.38
Final week	38.38 ± 0.62	41.13 ± 0.72	32.88 ± 0.46^****, ####^

*p*-values were expressed as ****p < 0.0001 vs. control group and ^####^*p* < 0.0001 vs. placebo group.
